# Puddle formation and persistent gaps across the non-mean-field breakdown of superconductivity in overdoped (Pb,Bi)_2_Sr_2_CuO_6+*δ*_

**DOI:** 10.1038/s41563-023-01497-1

**Published:** 2023-03-06

**Authors:** Willem O. Tromp, Tjerk Benschop, Jian-Feng Ge, Irene Battisti, Koen M. Bastiaans, Damianos Chatzopoulos, Amber H. M. Vervloet, Steef Smit, Erik van Heumen, Mark S. Golden, Yinkai Huang, Takeshi Kondo, Tsunehiro Takeuchi, Yi Yin, Jennifer E. Hoffman, Miguel Antonio Sulangi, Jan Zaanen, Milan P. Allan

**Affiliations:** 1 Leiden Institute of Physics, Leiden University, Leiden, The Netherlands; 2 Department of Quantum Nanoscience, Kavli Institute of Nanoscience, Delft University of Technology, Delft, The Netherlands; 3 Institute of Physics, University of Amsterdam, Amsterdam, The Netherlands; 4 QuSoft, Amsterdam, The Netherlands; 5 Institute for Solid State Physics, University of Tokyo, Kashiwa, Japan; 6 Toyota Technological Institute, Nagoya, Japan; 7 Zhejiang Province Key Laboratory of Quantum Technology and Device, Department of Physics, Zhejiang University, Hangzhou, China; 8 Collaborative Innovation Centre of Advanced Microstructures, Nanjing University, Nanjiang, China; 9 Department of Physics, Harvard University, Cambridge, MA USA; 10 Department of Physics, University of Florida, Gainesville, FL USA; 11 National Institute of Physics, College of Science, University of the Philippines, Diliman, Quezon City, Philippines; 12 Institute-Lorentz for Theoretical Physics, Leiden University, Leiden, The Netherlands

**Keywords:** Superconducting properties and materials, Electronic properties and materials

## Abstract

The cuprate high-temperature superconductors exhibit many unexplained electronic phases, but the superconductivity at high doping is often believed to be governed by conventional mean-field Bardeen–Cooper–Schrieffer theory^1^. However, it was shown that the superfluid density vanishes when the transition temperature goes to zero^2,3^, in contradiction to expectations from Bardeen–Cooper–Schrieffer theory. Our scanning tunnelling spectroscopy measurements in the overdoped regime of the (Pb,Bi)_2_Sr_2_CuO_6+*δ*_ high-temperature superconductor show that this is due to the emergence of nanoscale superconducting puddles in a metallic matrix^4,5^. Our measurements further reveal that this puddling is driven by gap filling instead of gap closing. The important implication is that it is not a diminishing pairing interaction that causes the breakdown of superconductivity. Unexpectedly, the measured gap-to-filling correlation also reveals that pair breaking by disorder does not play a dominant role and that the mechanism of superconductivity in overdoped cuprate superconductors is qualitatively different from conventional mean-field theory.

## Abstract (web summary)

The authors present scanning tunnelling spectroscopy data that show that the mechanism of superconductivity in overdoped cuprate superconductors is qualitatively different from conventional mean-field theory.

## Main

The essence of high-temperature superconductivity in the cuprates revolves around doping a Mott insulator. Superconductivity emerges when hole doping is greater than 5% per lattice site; the transition temperature *T*_c_ initially increases through the underdoped region of the phase diagram, before it decreases again in the overdoped region^1^. Superconductivity breaks down completely at roughly 27% doping. For the strongly overdoped region, it is often assumed that screening sufficiently reduces electron–electron correlations, enough for a Fermi liquid to appear^6–8^. The superconducting state is then of the Bardeen–Cooper–Schrieffer (BCS) type, and the suppression of superconductivity is a consequence of a diminishing pairing interaction. Evidence for such conventional behaviour in the overdoped regime comes from photoemission experiments, which suggest the existence of a full Fermi surface with superconductivity, as indicated by an energy gap that opens up in a BCS fashion below *T*_c_^9,10^. As a caveat, very recent magneto-transport experiments indicate that even at high doping, the normal state has strange metal features^11^.

The first surprise in this regard was the discovery that the superfluid density decreases linearly to zero with doping beyond optimal doping^2,3,12^, contrary to the BCS expectation that it should be of the order of the total carrier density and hence proportional to the doping level^1,2^. Additionally, optical conductivity measurements revealed a large density of metallic carriers below *T*_c_^12^, suggesting a filling of the superconducting gap due to pair breaking. One possible explanation for these observations involves potential disorder, reducing the electron mean free path, at length scales comparable to the small coherence length that is typical for the cuprates^4,5^. According to Bogoliubov–de Gennes (BdG) theory (that is, BCS in spatially heterogeneous systems), disorder at these length scales leads to emergent granular superconductivity^4,13–16^, that is, puddles of well-developed superconductivity with a size set by the coherence length, separated by regions where the gap is suppressed. The resulting weak-link superconductor will show a low superfluid density.

**Fig. 1:**
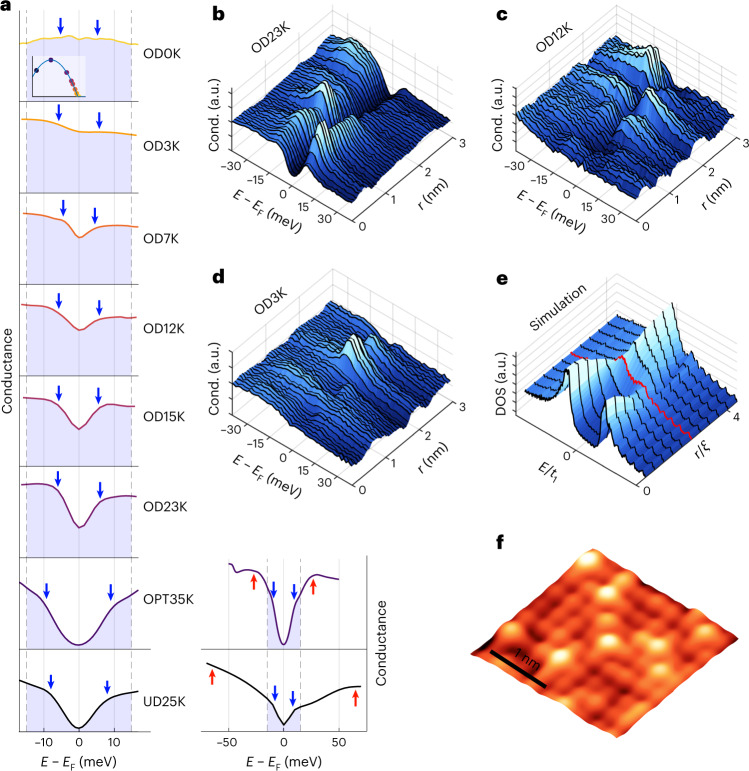
Heterogeneous gap filling in Bi2201. **a**, The average spectra of eight different doping levels, labelled by their *T*_c_; UD, underdoped; OPT, optimally doped; OD overdoped; *E*_F_, Fermi energy. The shaded areas indicate the energy range used in the fitting procedure. The blue arrows show the average extracted gap magnitude. For the UD25K and OPT35K samples, the red arrows in the righthand images indicate the pseudogap as determined by He et al.^32^. The inset in the top spectrum indicates the position of the samples on the superconducting dome. **b**–**d**, Spectra along 3 nm line-cut for the OD23K, OD12K and OD3K samples, respectively. These raw, unprocessed spectra indicate the high degree of electronic inhomogeneity in these samples. **e**, A line-cut of spectra from a self-consistent BdG simulation from the centre of a superconducting puddle (*r* = 0) to the metallic environment, which shows the van Hove singularity modelled to be close to the Fermi level (main text and Supplementary Section IV for details). The boundary of the puddle is indicated by the red spectrum. The pairing interaction is non-zero inside the puddle (that is, inside the red line) and is zero outside it. The energy unit is relative to the hopping parameter *t*_1_, and the length unit is relative to the coherence length *ξ* (Supplementary Section IV). DOS, density of states. **f**, Typical topography measured on the OD12K sample on the same length scale as **b**–**d**.

**Fig. 2:**
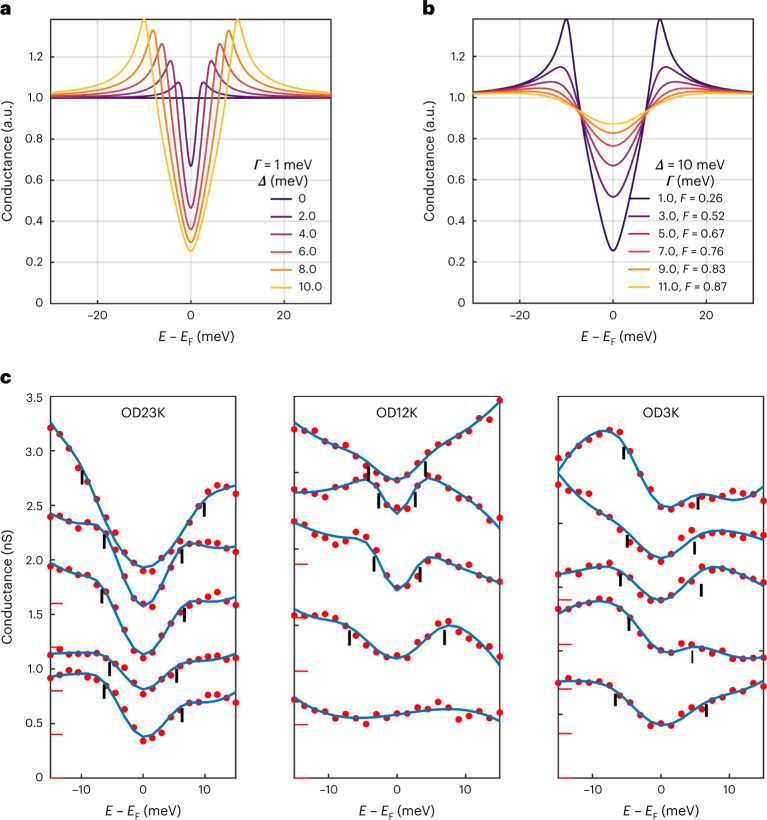
Gap filling versus gap closure. **a**,**b**, Difference between gap closure (**a**) and gap filling (**b**) by presenting a sweep of the gap magnitude parameter *Δ* for constant *Γ*, and a sweep of the scattering rate parameter *Γ* for constant *Δ*, respectively. **c**, Example fits from our model applied to our raw data for the OD23K, OD12K and OD3K data. The zeros of the spectra are offset for better visibility, as indicated by the red marks. The black marks indicate the gap width as determined by the model.

**Fig. 3:**
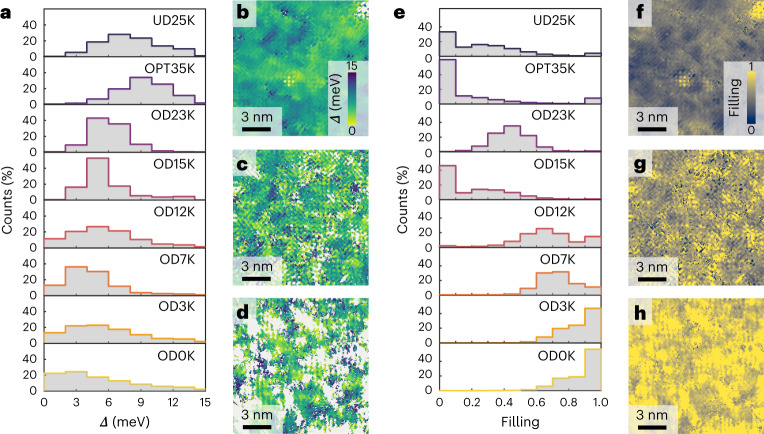
Doping dependence of the spatially resolved gap filling and gap magnitude. **a**, Gap magnitude histogram for each doping concentration. **b**–**d**, The spatial distributions of the gap magnitude for the OD23K, OD12K and OD3K samples, respectively. The spectra that are omitted from the histograms (main text and Supplementary Section II) are indicated by the white areas in **b**–**d**. **e**, Gap filling histogram for each doping level. **f**–**h**, Spatial distribution of the gap filling for the OD23K, OD12K and OD3K samples, respectively.

**Fig. 4:**
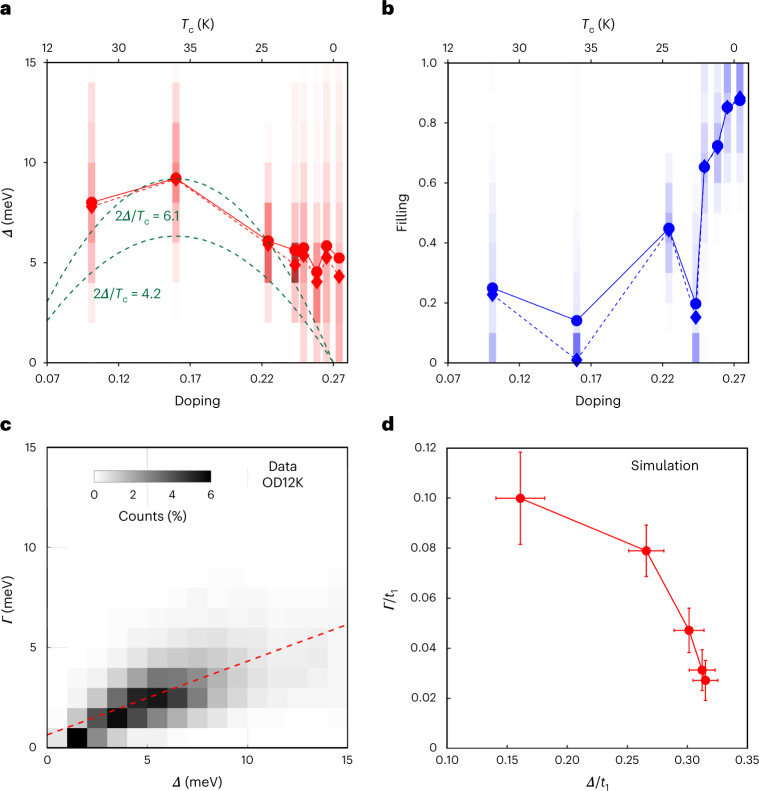
Gap-filling-driven breakdown of superconductivity and the contradiction to BdG. **a**,**b**, The mean (circles) and median (diamonds) of the gap magnitude (**a**) and the gap filling (**b**). The shaded areas represent the local variations in the gap magnitudes and fillings by depicting the histograms. The green dashed lines in **a** show the expectation of a gap size proportional to *T*_c_, with a proportionality constant either chosen to match the OPT35K data point, or determined by weakly coupled *d*-wave BCS theory. The doping levels were calculated using the Presland formula for the superconducting samples, and using the antinodal band shift measured by photoemission for the OD0K sample. **c**, Two-dimensional histogram of the measured local relation between gap magnitude *Δ* and pair-breaking parameter *Γ* for the OD12K sample. The positive correlation between the two is indicated by the red line. **d**, The relation between the gap magnitude and pair-breaking parameter extracted from self-consistent BdG simulations (Fig. 1e) using the same fitting model. In contrast to **c**, we find a clear negative correlation. The error bars indicate the uncertainty in the obtained values due to the fitting process (confidence intervals of the fit).

We investigate these issues using scanning tunnelling spectroscopy, which yields the spatial distribution of the electron density of states with atomic-scale precision. Our measurements show that in (Pb,Bi)_2_Sr_2_CuO_6+*δ*_ (Bi2201) which has one CuO_2_ layer per unit cell and is known to have a high residual resistivity^11^, such a ‘puddled’ superconductor does indeed develop at high doping (Figs. 1 and 2). The typical spatial extent of the puddles is a few nanometres, of the order of the small coherence length in this system (Fig. 3). Our measurements additionally reveal that the superconducting gap persists beyond the dome, and that instead the heterogeneity is driven by gap filling (Fig. 4). This strongly suggests that the breakdown of superconductivity is not a result of a vanishing pairing interaction. A comparison with BdG simulations suggests that this filling is likely due to the decay of the Cooper pairs in surrounding metallic areas, which in turn explains the observation of a large density of metallic carriers. Unexpectedly, we also find a striking violation of a basic BdG rule. Within BdG theory, pair breaking goes hand in hand with gap closing, because depletion of the number of Cooper pairs in a superconductor leads to a diminishment of the gap magnitude *∆* as well: ${\it{\varDelta }} = V\mathop {\sum}\nolimits_k {\left\langle {c_{k \uparrow }^ + c_{ - k \downarrow }^ + } \right\rangle }$, where *V* is the attractive interaction and *c*^+^ variables are electron field operators. Instead, our data show that the puddles characterized by the largest gap magnitudes also exhibit the largest gap filling (Fig. 4c), and that the average gap magnitudes barely depend on doping (Fig. 4a). We therefore conclude that the physics governing the superconducting transition is of a different, non-mean-field kind.

To arrive at these findings, we study a series of Bi2201 samples with eight different doping levels, from underdoped to beyond the superconductor–metal transition, with an emphasis on the strongly overdoped regime. We chose Bi2201 because it has only one CuO_2_ plane per unit cell, and has a rather large residual resistivity^17^, suggesting that disorder is exceptionally important. On each sample, we measure the atomic-scale-resolved differential conductance *g*(*E*,**r**) as a function of bias energy *E* and location **r**, which is proportional to the Bogoliubov quasiparticle density of states.

We first consider the spatially averaged *g*(*E*) spectra obtained at 4.2 K (Fig. 1a). Consistent with earlier reports^18,19^, crossing into the overdoped regime, the spectra acquire an increasingly large non-zero Bogoliubov quasiparticle density of states at the Fermi level. This is remarkable as this quantity should go to zero for a standard *d*-wave BCS superconductor, but it is consistent with results from optical conductivity measurements^12^. It remains to be seen whether angle-resolved photoemission spectroscopy (ARPES), if performed in strongly overdoped regions with such small gaps, would observe a similar phenomenology in both Bi2201 and (Pb,Bi)_2_Sr_2_CaCu_2_O_8__+__*δ*_ (Bi2212). We investigate this phenomenology using individual spectra, as in a heterogeneous situation like this, the average spectra do not represent the phenomenology adequately (Fig. 1b–d).

Next, we use a phenomenological model to fit all spectra over the whole doping range to extract the superconducting gap and gap filling of each individual spectrum. We calculate the spectral weight on each point **k** = (*k*_*x*_, *k*_*y*_) on the Fermi surface using a Dynes formula with superconducting gap *∆*_**k**_ = *∆*(cos(*k*_*x*_) − cos(*k*_*y*_))/2, where *∆* is the maximal gap, and then average over the Fermi surface^20^. We use the Dynes formula^21^ as a mere phenomenological description constructed to reveal characteristic scales for the observed gap size and the gap filling, and discuss interpretational concerns after the presentation of the data. Our model yields the following function for the modelled differential conductance:

1 $$\begin{array}{l}g\left( E \right) = P\left( E \right) \times \left\langle {{{{\mathrm{Dynes}}}}\left( {E,{\it{\varDelta }}_{{{{{\mathbf{k}}}}}},{\it{\varGamma }}} \right)} \right\rangle _{{{{\mathrm{FS}}}}} \\= P\left( E \right) \times \left\langle {{{{\mathrm{Re}}}}\left( {\frac{{E + i{\it{\varGamma }}}}{{\sqrt {\left( {E + i{\it{\varGamma }}} \right)^2 - \varDelta _{{{\mathbf{k}}}}^2} }}} \right)} \right\rangle _{{{{\mathrm{FS}}}}},\end{array}$$

where 〈〉_FS_ indicates the average over the Fermi surface, *P*(*E*) is a third-degree polynomial function to account for background variation and Dynes(*E*, *∆*_**k**_, *Γ*) is the Dynes function with the pair-breaking parameter *Γ*. For this study, we concentrate on the superconducting gap and thus restrict ourselves to a ± 15 meV range. (In the underdoped and optimally doped range, which are not the focus of this paper, a pseudogap exists at a larger energy scale, as indicated by the red arrows in Fig. 1a). Lastly, we convolute *g*(*E*) with a Gaussian function to account for spectrum broadening due to a finite temperature and the lock-in modulation.

We define the filling parameter *F* as the ratio *g*(*E* = 0,*T* → 0)/*P*(*E* = 0), which can be expressed in terms of our fitting parameters as:

2 $$F = \left\langle {1/\sqrt {1 + \left( {{\it{\varDelta }}_{\bf{k}}/{\it{\varGamma }}} \right)^2} } \right\rangle _{{\mathrm{FS}}}.$$

Figure 2a,b illustrates how the model differentiates between gap closure, controlled by *∆*, and gap filling, controlled by *Γ* or *F*. Figure 2c shows some typical spectra and fits from various locations. It is clear that, when compared to the scenarios presented in Fig. 2a,b, the measured spectra look more similar to the filling scenario as opposed to the closing scenario. We then fit roughly 10^5^ spectra from eight different doping levels with this model and display the extracted gap size and gap filling in Fig. 3. We note that for the strongly overdoped samples, high a signal-to-noise ratio is key for successful fits; the traces shown in Figs. 1b–d and 2c are raw spectra without any averaging. A further challenge is that at higher doping, a substantial fraction of spectra exhibit completely gapless regions. We identify such spectra after fitting and exclude them from subsequent analysis. In the Supplementary Information, we provide details (Supplementary Section IIB and Supplementary Fig. 4) and demonstrate that our key results are independent of these choices (Supplementary Section IIA–C). We also provide a modified version of the model with an alternative definition of the gap filling and show that our results are independent of the precise definition of gap filling (Supplementary Section IID).

We start our discussion with the spatial maps of the gap size *∆*(**r**) as a function of doping (Fig. 3b–d). Strikingly, while more spectra are fully filled at higher doping, the average gap size remains roughly constant on the strongly overdoped side (Fig. 3a). Initially, the gap size increases when moving from underdoped to optimally doped. Beyond optimal doping, the gap size barely decreases anymore when going through the overdoped and strongly overdoped side, and instead remains roughly constant—even beyond the superconductor-to-metal transition. In particular, throughout the strongly overdoped region, we observe an almost constant average gap amplitude even though *T*_c_ is rapidly decreasing. Our study thus excludes a homogeneously diminishing pairing interaction as the cause of the superconductor-to-metal transition.

Given a constant gap, what drives the changes in spectra on the overdoped side? Our analysis indicates that it is the gap filling. We extract the gap filling, *F*, using equation (2), for each measured sample, and present the distribution of the gap fillings in Fig. 3f–h and their histograms in Fig. 3e. Remarkably, the mean gap filling changes considerably over the doping range. In the optimally doped region, the spectra have a filling close to zero; that is, they are fully gapped. Crossing into the overdoped regime, a subset of spectra starts to develop a finite gap filling. This subset grows with further doping, with all spectra having a finite filling in the strongly overdoped regime. The values of *F* shift markedly in this doping regime from nearly fully gapped (*F* = 0) near optimal doping to almost fully filled (*F* = 1) towards the strongly overdoped regime and extending into the metallic regime. The trends in gap closing and gap filling are summarized in Fig. 4a,b: as the doping is increased into the overdoped regime, the gap size remains roughly constant; by contrast, the gap filling increases rapidly. Thus, a first key result of this paper is that it is not a decaying gap width *Δ*, but an increasing gap filling *F* that is responsible for the diminishing superconductivity and that eventually drives the superconductor-to-metal transition. We also note that the persistence of the superconducting gap we observe is remarkably similar to the persistent spin gap observed in a similar doping range^22^.

Notably, the gap filling is highly heterogeneous, as can be seen from the width of the distributions in Fig. 3e and in the spatial maps in Fig. 3f–h. We observe areas both with and without a gap, each existing at a length scale consistent with the coherence length (~1.5 nm). Some spectra exhibit a peak that can be associated with a van Hove singularity, as reported previously^18,19^, but we note that it is a highly anomalous one: both the energy and the amplitude of the peak vary in space on length scales that are not consistent with the spatially averaged antinodal signature observed in photoemission^23^. Further, we find this peak only in the strongly overdoped regime, whereas ARPES measurements suggest that the van Hove singularity should be observable in lower-doped samples as well, at energies still easily measurable by scanning tunnelling microscopy. The question of the van Hove singularity in scanning tunnelling microscopy data remains open. Notwithstanding the van Hove singularity, our observations indicate that the breakdown of superconductivity in the overdoped regime of the single-layer bismuth cuprate is likely caused by an emergent strongly inhomogeneous superconductivity, leading to an effective weak-link physics that explains the diminishing superfluid density. Hence, at first glance, our data suggest that theoretical models involving the disorder-driven breakdown of superconductivity in the BdG framework^4,5,13,14,16^ are a good description of the physics of strongly overdoped Bi2201, with the additional information that it is the gap filling that drives the formation of the superconducting puddles.

Next, we focus on the origin of the gap filling. According to BdG theory, the excitations that fill the gap are quasiparticles of the Fermi liquid normal state that are released by breaking up Cooper pairs. Well-known causes for pair breaking are potential disorder^4,5^ (for a *d*-wave superconductor) and thermal phase fluctuations^24–26^. However, if potential disorder were the only culprit, the areas where the pair breaking is smallest (where superconductivity survives best) should have the largest gaps, which is not what we observe. We demonstrate this in Fig. 4c, where we show the local relationship between the gap size *Δ* and the pair-breaking *Γ*, and find a clear positive correlation between the two. Further, we can exclude thermal phase fluctuations based on our temperature-dependent measurements, up to 20 K for the overdoped sample with *T*_c_ = 9 K (OD9K). Thermal phase fluctuations should lead to a strongly temperature-dependent filling, in contrast to our observations (Supplementary Section III).

We therefore consider an alternative candidate for pair breaking: the decay of Cooper pairs into smaller gap or metallic regions, as previously suggested^27,28^. This can be seen as akin to an inverse proximity effect^29^. We are not aware of self-consistent simulations for this scenario in the literature, but they are possible with state-of-the-art numerical methods. We start with a large real-space supercell implementing a realistic tight-binding band structure. We then introduce the superconducting puddles by switching on a local pairing interaction characterized by a linear dimension *L* that is approaching the (bulk) coherence length. The BdG equations are then solved self-consistently (Supplementary Section IV for further details), and typical outcomes are shown in Fig. 1e. The simulated spectra are surprisingly similar to the experimental ones, and one might wonder whether this gap-filling-dominated physics is connected to certain disordered superconductors^15,30^ and interface superconductors^31^ with a local density of states phenomenology that is not dissimilar from what we observe here.

However, there is one aspect of our data that is markedly inconsistent with the BdG description of granular superconductors. Our data show that the largest gaps also exhibit the strongest gap filling (Fig. 4c), while within BdG, gap filling should always go hand in hand with a decrease of the gap magnitude. Our self-consistent simulations confirm that this is indeed also valid for the heterogeneous case: upon application of our fitting model to the calculated spectra shown in Fig. 1e, we find that the regions with the largest gaps show the least amount of pair breaking, as shown in Fig. 4d. Self-consistency in the calculations is necessary here; fixing the gap magnitude artificially would obscure any effect from pair breaking on the gap magnitude. The comparison in Fig. 4c,d shows a striking inconsistency between the experiment and BdG expectation.

In summary, our real-space imaging reveals a strongly heterogeneous superconductivity consisting of superconducting puddles with a size set by the coherence length immersed in a metallic matrix. This explains the diminishing superfluid density^2,3^ and the origin of the large fraction of metallic carriers^12^: it stems from the filling of the gap. Our data further demonstrate that superconductivity does not, as it is often assumed, become conventional in the strongly overdoped regime. The breakdown of superconductivity is not the consequence of a vanishing pairing interaction and does not follow the BdG description. Furthermore, the gap filling is entirely different from simple quasiparticles populating the gap and counting the number of broken BCS Cooper pairs. Instead, what fills the gap might be related to the strange normal state^11^, for example, collective excitations of an unknown kind rooted in the ‘strange metal’ physics, which at present cannot be calculated, or to electrons from a different sector, but not by means of simple pair breaking. Last but not least, this unconventional physics may not be limited to the low-temperature, overdoped regime. Scanning tunnelling microscopy studies at an optimal doping of Bi2212 showed a rather similar puddling effect upon approaching the superconducting transition temperature^27,28^. This may imply that the physics of the thermal transition—the ‘high *T*_c_’ problem itself—is governed by unknown physics. Therefore, it would be interesting to revisit the high-temperature regime in Bi2212 to make this more precise.

## Online content

Any methods, additional references, Nature Portfolio reporting summaries, source data, extended data, supplementary information, acknowledgements, peer review information; details of author contributions and competing interests; and statements of data and code availability are available at 10.1038/s41563-023-01497-1.

## Supplementary information

Supplementary Information Supplementary sections I–VII (figs. 1–14 and discussion).

## Data Availability

The full three-dimensional datasets used for this paper, and the data used to generate Figs. 1–4 and Supplementary Figs. 1–13, will be available on Zenodo, 10.5281/zenodo.7643856.

## Code availability

The code used for fitting and generating figures is available upon reasonable request to the authors.
