# Supplementary material for: Puddle formation and persistent gaps across the non-mean-field breakdown of superconductivity in overdoped (Pb,Bi)2Sr2CuO6+δ
Source: Nat Mater. 2023 Mar 6;22(6):703–9. doi: 10.1038/s41563-023-01497-1 (PMC10234808; doi:10.1038/s41563-023-01497-1)
Supplement: Supplementary file 1 — Supplementary sections I–VII (figs. 1–14 and discussion). [file 41563_2023_1497_MOESM1_ESM.pdf]

nature materials

Letter https://doi.org/10.1038/s41563-023-01497-1

Puddle formation and persistent gaps across the non-mean-field breakdown of superconductivity in overdoped $(Pb,Bi)_2Sr_2CuO_{6+\delta}$

In the format provided by the authors and unedited

Supplementary Information

I. Experimental Methods

We performed a systematic study on a series of $(Pb,Bi)_2Sr_2CuO_{6+\delta}$ samples with 8 distinct doping levels, covering the range from underdoped (UD25K) to strongly overdoped (OD0K) side. Single crystal samples were grown by the conventional floating-zone technique[1,2]. The UD25K, OP35K, and OD15K samples contain La doping, i.e. $(Pb,Bi)_2(La,Sr)_2CuO_{6+\delta}$, while the rest of the samples are without La doping. The doping levels, transition temperatures T_c, and measurement temperatures are listed in Table S1. The doping levels of the superconducting samples are determined using the Presland formula, while the doping level of the OD0K sample is extracted from the rigid band shift measured by ARPES (see supplementary section V). All samples were cleaved *in situ* in a cryogenic environment and inserted immediately into the STM. The data were acquired using different home-built cryogenic STMs among three groups.

Name	Doping	*T*c (K)	Measurement *T* (K)	Data acquired by	Samples fabricated by	V_{setup} (mV)/ I_{setup} (pA)/ Lock-in Amplitude (mV)	Effective Energy Resolution (FWHM, meV)
UD25K	0.101	25	5.7	Hudson group	Kondo/Takeuchi group	-100/400/1	2.7
OP35K	0.160	35	5.7	Hudson group	Kondo/Takeuchi group	-100/400/1	2.7
OD23K	0.224	23	4.2	Allan group	UvA group	-150/150/1.5	2.58
OD15K	0.243	15	6	Hoffman group	Kondo/Takeuchi group	-100/100 /2	3.5
OD12K	0.249	12	4.2	Allan group	UvA group	-200/170/1.5	2.58
OD9K	0.255	9	4.2 – 20	Allan group	UvA group	-150/200/1.5	2.58 – 6.42
OD7K	0.258	7	2.2	Allan group	UvA group	-20/600/1.5	2.50
OD3K	0.265	3	4.2	Allan group	UvA group	-200/170/1.5	2.58
OD0K	0.274		4.2	Allan group	UvA group	-25/200/1.5	2.58

Table 1: Samples and their measurement conditions in this study.

II. The phenomenological model to fit spectra

A. The *d*-wave gap model

The *d*-wave gap is here modeled as a mean of multiple *s*-wave gaps, one for each point along the Fermi Surface. For each *s*-wave gap, the gap size $\Delta_{\mathbf{k}}$ is given by $\Delta_{\mathbf{k}} = \Delta(\cos k_x - \cos k_y)/2$. The points k_x, k_y are found using the Fermi Surface of the tight-binding model for the OD15K sample[1] although the final shape of the spectrum varies a lot with the exact points in k-space used. Each s-wave gap is generated by using the Dynes formula

$$\text{Dynes}(E, \Delta_{\mathbf{k}}, \Gamma) = \text{Re}\left(\frac{E+i\Gamma}{\sqrt{(E+i\Gamma)^2-\Delta_{\mathbf{k}}^2}}\right),$$

where the same Γ is used for all *s*-wave gaps. The resulting *d*-wave gap is the mean of all *s*-wave gaps. To account for the normal-state density of states (DOS), the *d*-wave gap function is multiplied with a polynomial function, typically of the 3rd order. The resulting spectrum is then convoluted with a Gaussian function with a full width at half maximum (FWHM) given in the table above, in order to emulate the effect of finite temperature and the lock-in modulation have on the shape of the measured spectrum.

The points in momentum space are calculated only once before the fitting process to reduce computation time. The fitting parameters characterizing the gap are only Δ and Γ. To calculate the filling for the *d*-wave gap we calculate the mean of the filling for each individual *s*-wave gap using $F = [1 + (\Delta/\Gamma)^2]^{-1/2}$.

B. Statistical analysis with and without the excluded spectra

In the main text we "white out" certain spectra (white areas in Figs. 3b-d), and exclude them for the statistical analysis when either of the two conditions is met in the fit results: 1) $\Gamma > 20$ meV; 2) $\Delta > 15$ meV. Our interpretation is that these spectra are fully filled, for the following reason: the spectra that meet the first criterion have so much broadening that there is no well-defined gap. Similarly, for the spectra that meet the second criterion, the large "gap" is a reflection of background modulations. Such spectra are thus counted as fully filled. Still, we show here that including these spectra in the analysis does not alter our main conclusions.

Figure S1 shows, from left to right, the spatial distributions of: Δ, including "whited-out" (1st column) and excluding "whited-out" (2nd column) spectra, Γ, with (3rd column) and without (4th column) "whited-out" spectra, and F, the filling (5th column) for all samples. The images are ordered top to bottom, from lowest to highest doping, respectively. Following the argumentation in the preceding paragraph, the filling for "whited-out" spectra is set to 1, when they are included in the statistics. Figure S2 shows the histograms for Δ including "whited-out" spectra, and the histograms of Γ, both with and without "whited-out" spectra. The remaining histograms of Δ excluding the "whited-out" spectra and of the filling can be found in the main text. The histograms in Fig. S2 are summarized in Fig. S3 in a similar fashion to Fig. 4 of the main text.

From the spatial distributions and histograms of Δ and Γ in Figs S1-S3, we conclude that even when the "whited-out" spectra are considered: 1) the gap size still deviates from the $\Delta \propto T_C$ behavior in the OD regime; 2) gapped spectra can still be found in the non-SC sample in significant quantities.

The spatial averages of the "whited-out" spectra and of the rest spectra are shown in Fig. S4 for each sample. We find that after whiting out all samples, even the non-SC sample, show a gap in their average spectrum. We note that the spatially-averaged "whited-out" spectra in the OD regime are fully filled, i.e. they no longer have a gap, and often show a peak near Fermi level. This further justifies our choice to assign all these spectra a filling of 1. For the UD25K and OPT35K samples, the "whited-out" spectra are made up of spectra for which the fit has failed due to limited signal-to-noise. Even though these spectra appear as gapped, we attribute this to the presence of a pseudogap. We find that the assignment of $F = 1$ to these spectra does not alter the main conclusion either, given the relatively small portions of "whited-out" spectra in these samples (see Fig. S4). The increase of the area of "whited out spectra" in the OD as shown in Fig. S4 reaffirms the increased gap filling in these samples.

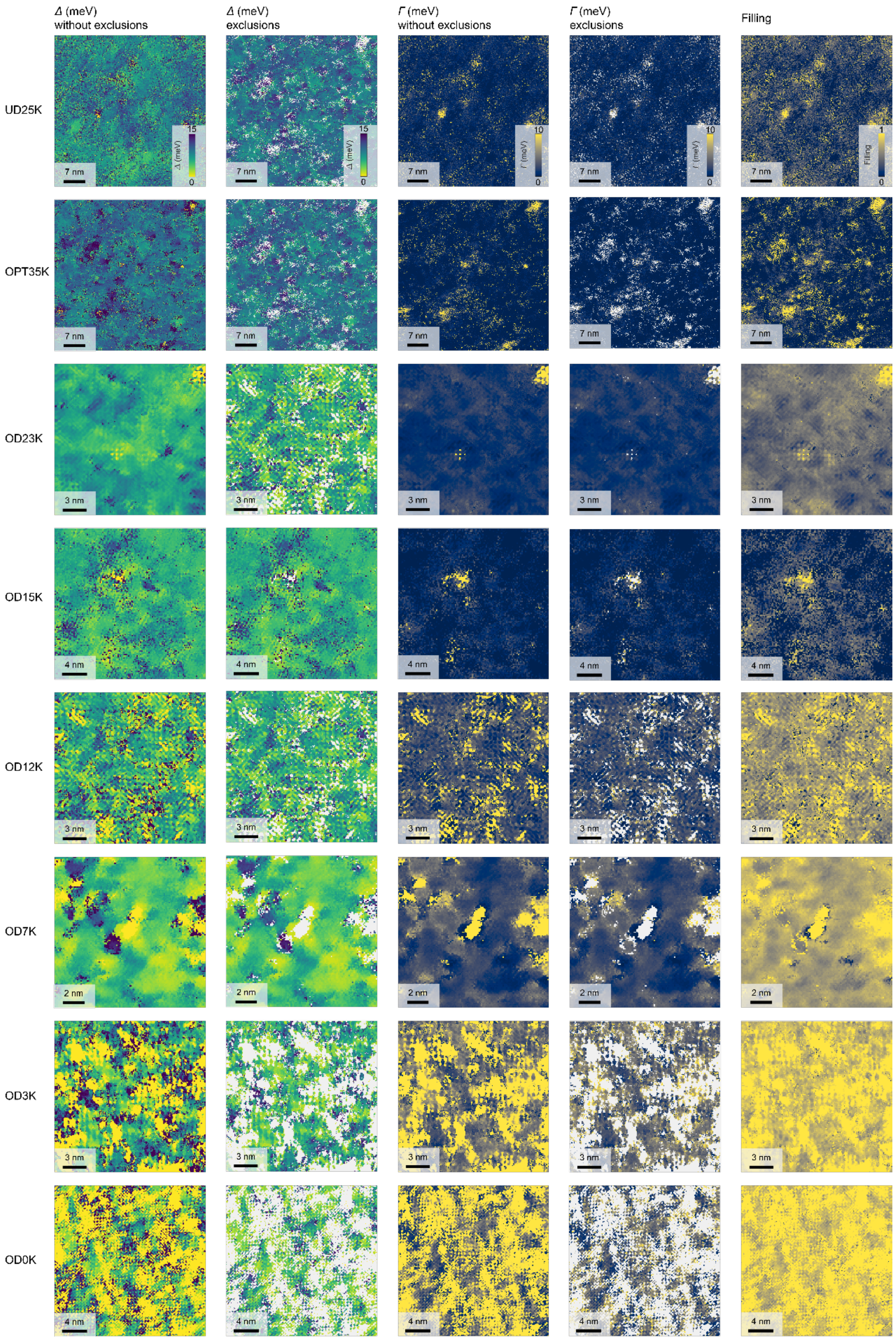

Figure S1: Spatial variations of the gap size *Δ* with and without exclusions, parameter *Γ* with and without exclusions, and the calculated filling for all samples. See text for details.

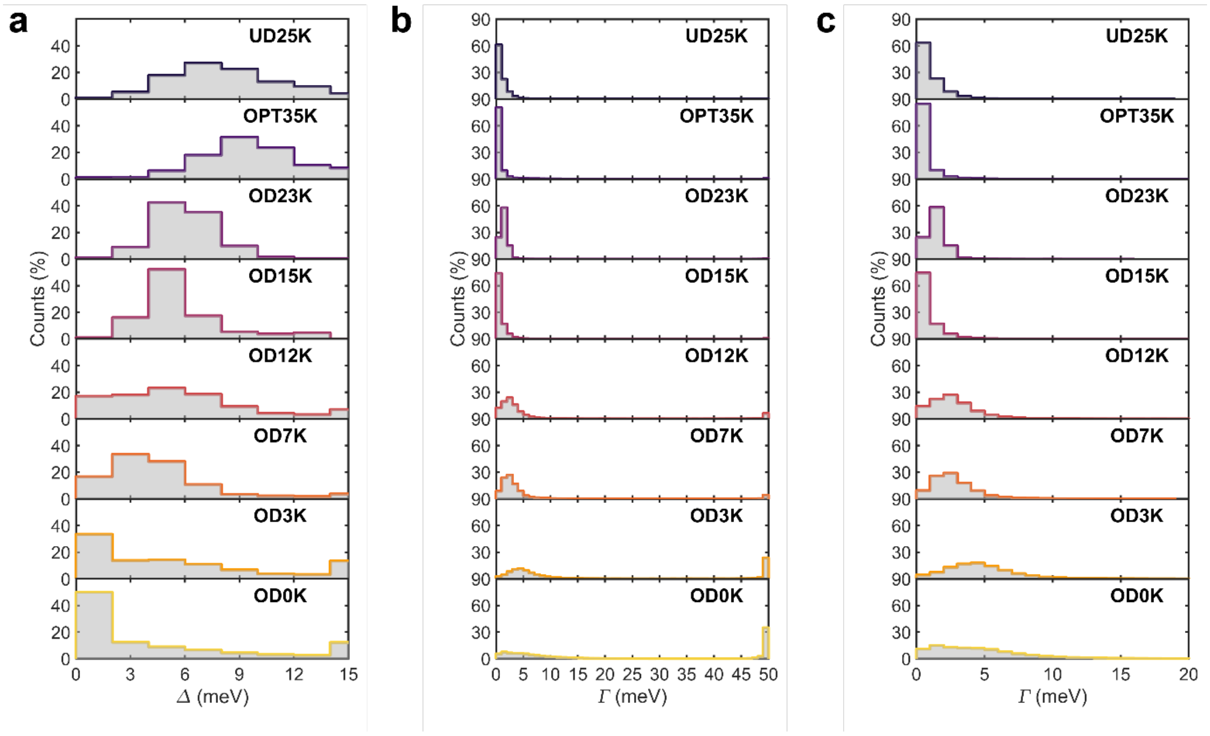

Figure S2: Histograms of the gap size *Δ* *without* any spectra that are whited out (a). Histograms for *Γ* including (b) and excluding whited-out spectra (c). See text for details.

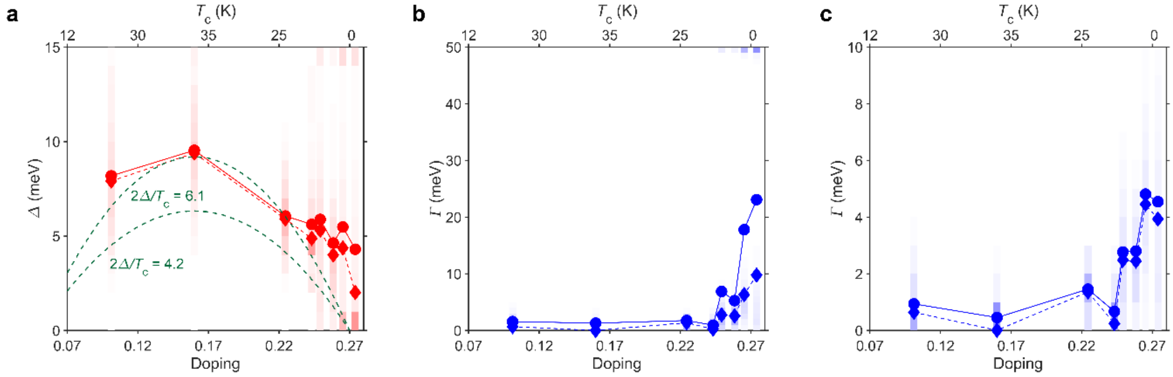

Figure S3: The results for *Δ* without excluding any spectra, and for *Γ* including and excluding "whited-out" spectra are summarized(a, b, c respectively). The circles indicate the mean *Δ*, *Γ* for each sample, with the diamonds indicating the medians. The shaded areas in the background represent the spread in values these parameters have. The green dashed lines in the left figure indicate the behavior expected for *Δ* proportional to T_c. The value of $2\Delta/T_c$ corresponds to the dirty d-wave BCS limit, while $2\Delta/T_c$ is chosen such that it matches the OPT35K data point.

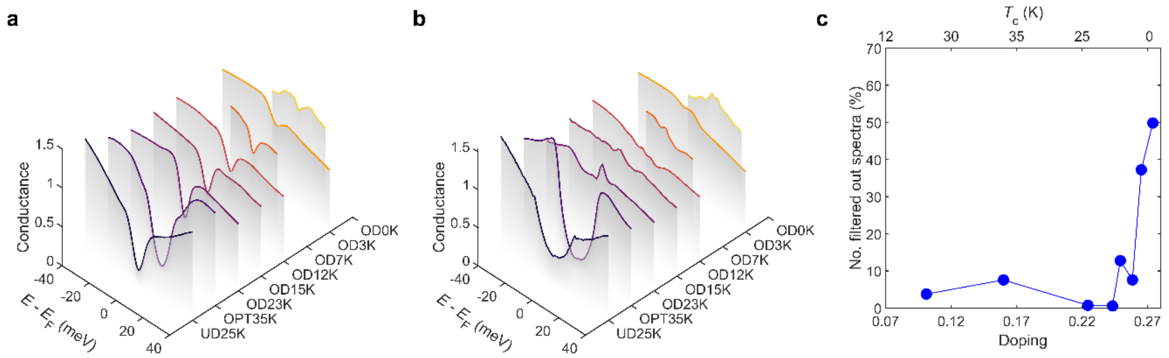

Figure S4: a) The spatially-averaged spectra after the "whited-out" spectra have been removed. b) Spatial averages of the "whited-out" spectra for each doping level. c) The proportions of "whited-out" spectra that make up each sample as a function of doping.

C. Fit parameters figure 2c

Sample	Spectrum	*Δ* (meV)	*Γ* (meV)
OD23K	1	6.3 ± 1.3	1.6 ± 0.7
	2	5.4 ± 1.3	2.1 ± 0.9
	3	6.7 ± 0.8	1.1 ± 0.4
	4	6.3 ± 0.5	0.9 ± 0.3
	5	9.9 ± 1.9	2.0 ± 0.5
OD12K	1	0.8 ± 2E5*	47 ± 5E6*
	2	6.9 ± 2.0	3.0 ± 1.0
	3	3.4 ± 0.8	1.1 ± 0.8
	4	2.7 ± 0.7	0.9 ± 0.7
	5	4.2 ± 1.7	2.9 ± 1.6
OD3K	1	6.7 ± 1.7	3.1 ± 0.9
	2	4.7 ± 1.1	2.9 ± 0.9
	3	5.9 ± 1.4	3.2 ± 0.9
	4	4.9 ± 2.7	3.2 ± 2.1
	5	5.4 ± 1.4	2.6 ± 0.9

* Spectra that do not show a gap and therefore have ill-defined gap parameters.

Table 2: Fitted values of *Δ* and *Γ* for the example spectra shown in fig 2c of the main text, together with the 95% confidence interval for those values. The spectra are numbered from bottom to top.

In table 2, we present the values of *Δ* and *Γ* as determined by our model for the examples shown in figure 2c of the main text. Also indicated are the 95% confidence intervals for those values. All confidence intervals are below the effective energy resolution of the experiment (see table 1), with

one exception. Spectrum number 1 of the OD12K sample does not show a gap, and therefore the values for Δ and Γ are ill-determined, as expressed by the confidence interval. This spectrum however also meets our conditions defined in SI IIB. As such, the poorly determined values for Δ and Γ are a clear indication that we in those cases we are dealing with spectra for which those parameters have no physical meaning.

D. Energy range for fitting and approximations for the normal density of states

The spectra in the UD and OPT samples show clear pseudogap (PG) features, with PG sizes ranging from 20 meV to over 60 meV (see main text, figure 1a). Furthermore, in the OD regime, the normal-state DOS shows a peak near the Fermi level. These additional features next to the superconducting gap complicate the accurate fitting of the superconducting gap. We circumvent this complication by limiting our analysis to a small window E_{win} around the Fermi level. In this reduced energy window, the additional features are only partly visible, and can be sufficiently approximated by a polynomial DOS. The choices of E_{win} and the order of the polynomial background are arbitrary but necessary choices made before the fitting procedure. Here we show the influence the particular choices have on the superconducting gap size and filling in the OD samples.

In the main text, we use E_{win} = ±15 meV, which is a choice made before the fitting procedure. Altering this choice does not affect the main conclusions of our analysis, as shown in figure S5. We repeat the analysis using different energy windows and find that the qualitative behavior does not change: Δ remains constant while F sharply increases in the SOD regime. Further increasing E_{win} beyond 20 meV, the highly inhomogeneous normal-state DOS becomes more significant, defeating the aim of focusing on the superconducting gap through an energy window. With an energy window smaller than 10 meV, we find that too little of a spectrum is left to characterize the superconducting gap accurately.

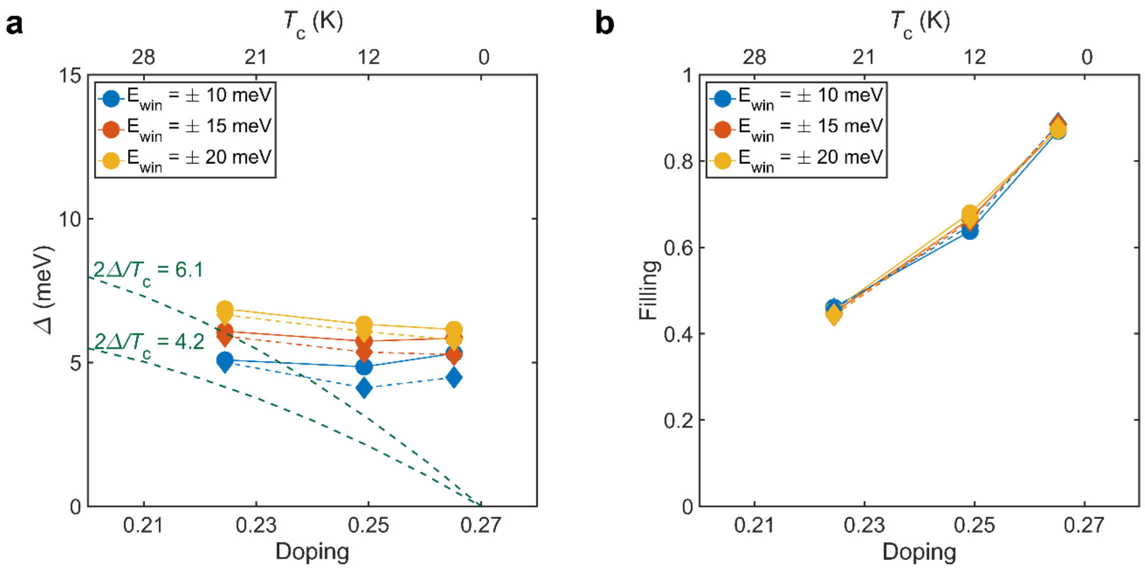

Figure S5: Dependence of our conclusion on the choice of the energy window. The dependencies on the fitting energy window for the average Δ and filling (a, b respectively) for the overdoped samples OD23K, OD12K, and OD3K. While the absolute values of the averages vary slightly with the cutoff energy, the overall behavior of a constant gap size and increasing filling factor is independent of the cutoff.

Another possible influential choice in the fitting procedure is the order of the background polynomial used to model the normal-state DOS. Figure S6 shows the mean gap size and filling for the OD23K, OD12K, and OD3K samples for different orders of polynomial ranging from 1st to 4th order. The overall behavior of nearly constant gap size and increasing filling is present for all polynomial orders. We opt to use a 3rd order polynomial in the main text as it offers the best balance between underfitting and overfitting.

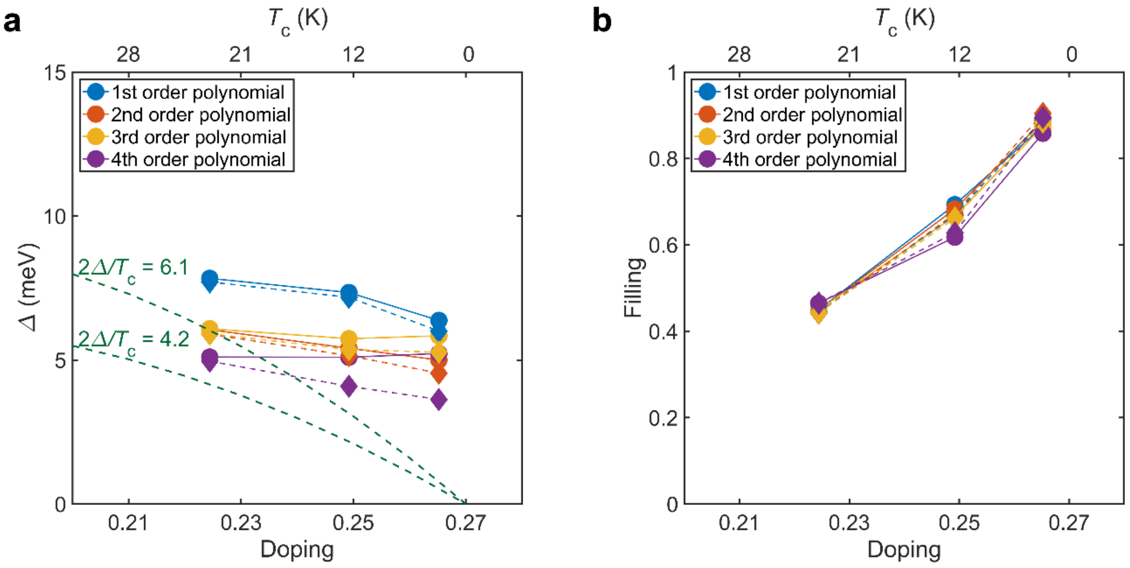

Figure S6: Dependence of our conclusion on the choice of the polynomial normal-state DOS. The average gap size and filling (a, b) for the overdoped samples OD23K, OD12K, and OD3K as the order of the polynomial normal-state DOS is varied. While the absolute values of the averages vary a bit among the various polynomials, the overall qualitative behavior of a barely varying gap size and the drastic increasing filling is present in all cases.

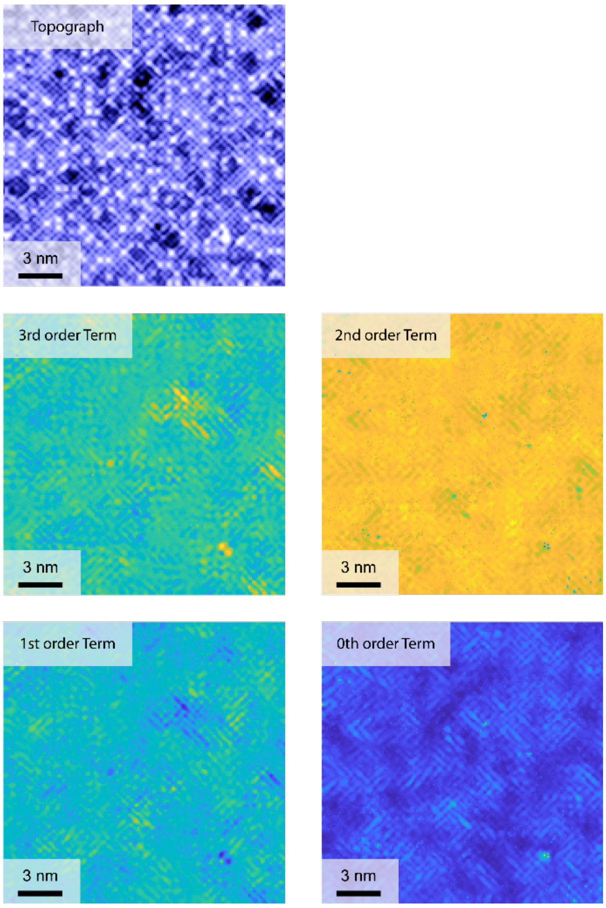

Figure S7: Topography of the OD12K, together with the spatial distributions of the polynomial background terms used in the fits in the main text. The correlation coefficients of the 3rd, 2nd, and 1st order terms with the topography are 0.08, -0.11, and -0.13 respectively, meaning they do not correlate with the topography. The 0th order term (i.e. the constant term) does correlate with the topography (coefficient of 0.37). The underlying relation of this is that they both relate to the same quantity, the average LDOS of that spectrum. If this is low the constant background term will go down. A low average DOS also means there are less states to tunnel into with our STM tip, meaning that the tip will move closer to the sample to maintain the same setup current. Hence, this location will register as a suppression of the surface. This is clearly visible when comparing the topography with the spatial distribution of the constant background term. Darker regions of the topography are also visible as darker regions of the constant term.

E. An alternative model

Here we introduce an alternative approach to determining the gap filling, and show that the conclusions are the same using this model. We use a model which explicitly includes the filling *F* as a fitting parameter, in contrast to extracting F using fit parameters Δ and Γ in the main text:

$$g(E) = P(E) * [(1 - \mathrm{F}) * \mathrm{Dynes}(\Delta, \Gamma) + \mathrm{F}],$$

where $P(E)$ and $\mathrm{Dynes}(\Delta, \Gamma)$ are defined the same as those in the main text. The gap filling is now explicitly parametrized by the parameter *F*, with *F* = 0 corresponding to fully gapped and *F* = 1 to fully filled. To prevent overfitting and to limit built-in correlations between fit parameters we fix the value of Γ. Figure S8 shows the average gap size and gap filling from the fit results using this model,

analogous to Fig. 4 in the main text. For this alternative model, we exclude spectra with a) Δ close to 0 (Δ < 1 meV), and b) F close to 1 (F > 0.95) from further analysis. In case a) the gap sizes become smaller than our thermally limited energy resolution, preventing an accurate determination of Δ. In case b) F becomes ill-defined as F can be absorbed into $P(E)$ when the gap is barely present. Fitting our data with this model, we find that the gap size remains constant in the OD regime, while the gap filling increases rapidly. Confirmation by an alternative model further strengthens the conclusions of the main text.

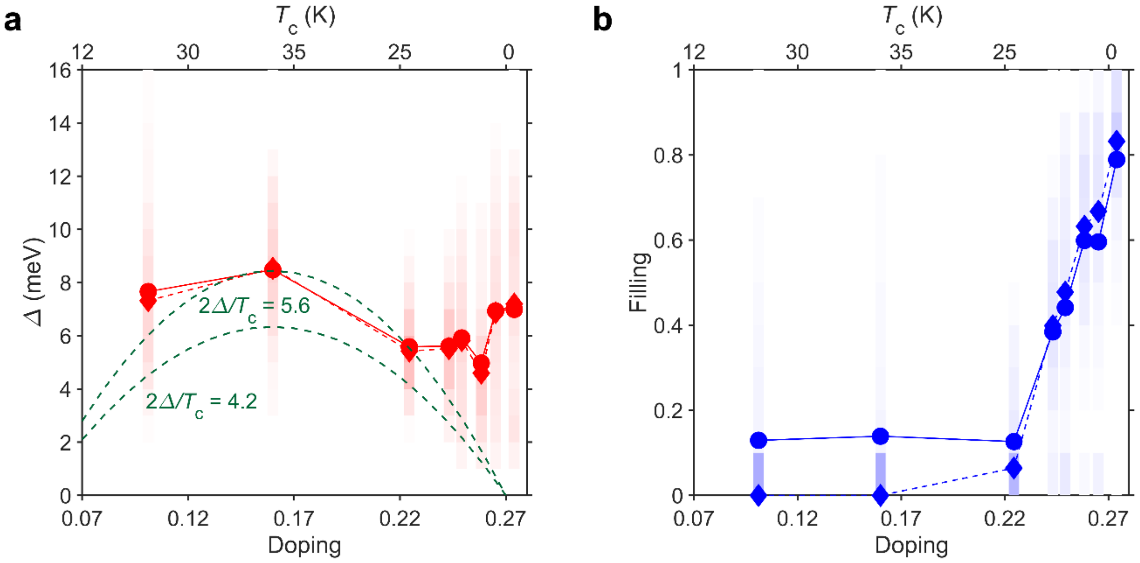

Figure S8 Gap magnitude and filling versus doping using the alternative model. The gap size (a) and the gap filling (b) as determined by the application of the alternative model described above. The shaded areas indicate the histograms of the parameters at each doping level. The average gap sizes and average filling are indicated by the circles. The median (diamonds) is shown to better reflect highly asymmetric distributions as is the case for the gap filling. The robustness of the qualitative trends against the use of different models reinforces the conclusions of the main text.

III. Temperature dependence

In figure S9a, we show the temperature evolution of the average spectrum measured in the same field of view on the OD9K sample. Furthermore, we show the median values for the gap and filling parameters as a function of temperature in the same field of view in Figures S9b and S9c, respectively. We find that a gap is still present up to 20K for the OD9K sample, even when the temperature-limited and lock-in broadened energy resolution is taken into account. With increasing temperature, we see that the gap magnitude and gap filling remain fairly constant up to 20K.

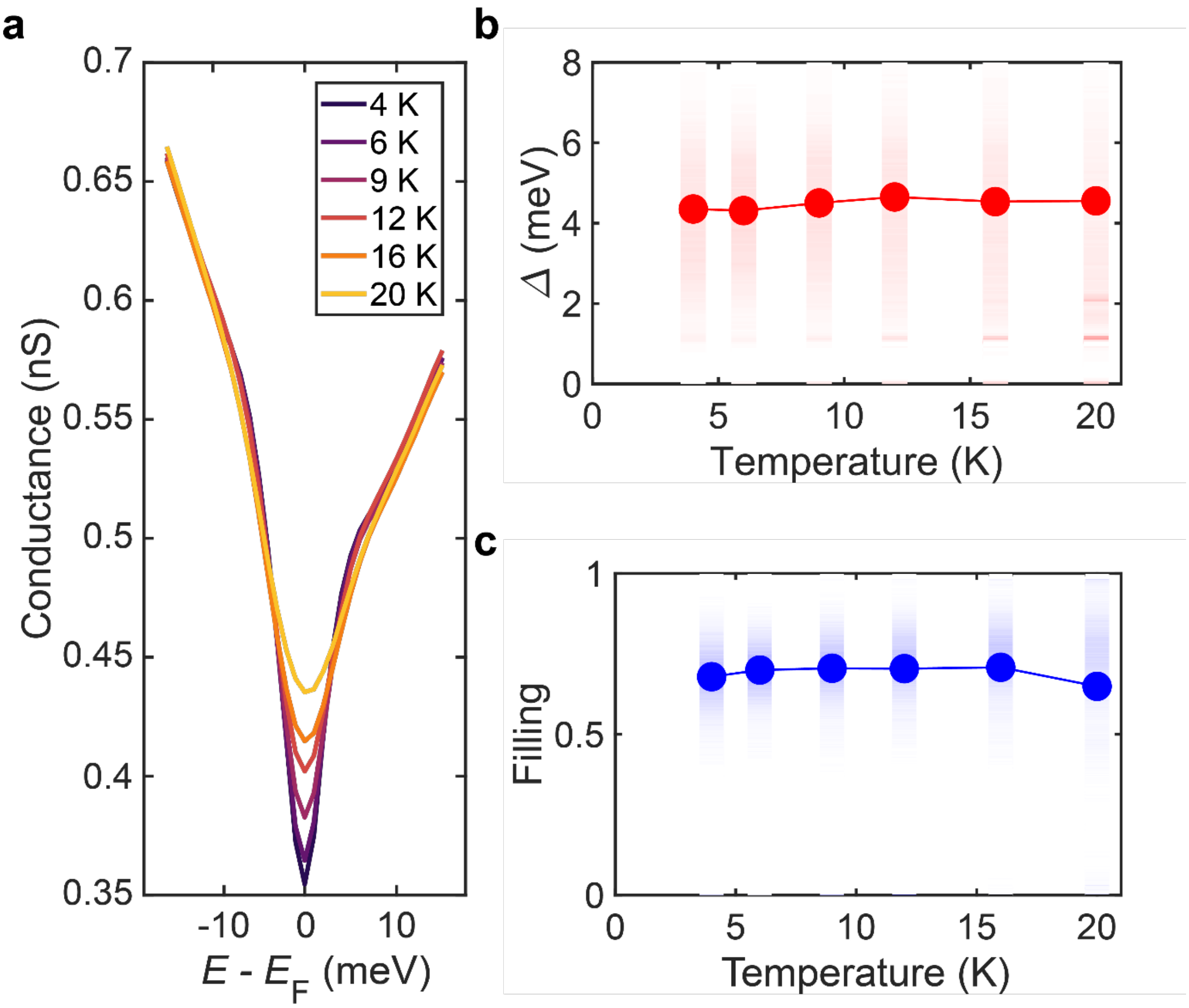

Figure S9: Temperature evolution of the gap width and gap filling. a) Average spectra measured in the same field of view on the OD9K sample (V = -150 mV, I = -200 pA). b) Median values for the gap magnitude in the same field of view of A, as a function of temperature. The shaded areas indicate the histograms of the gap at each temperature. c) Median values for the Filling parameter in the same field of view of A, as a function of temperature. The shaded areas indicate the histograms of the Filling at each temperature.

IV. Intrinsic Metal-Induced Pair-Breaking Effects Within a Superconducting Puddle Embedded in a Metallic Matrix

In this section, we consider the case of a small *d*-wave superconducting puddle whose size is of the order of the superconducting coherence length ξ_0, embedded in a metallic matrix. This is a particularly relevant model for the strongly overdoped regime. Our treatment of this system is entirely mean-field; we employ large-scale numerical simulations of the Bogoliubov-de Gennes Hamiltonian to uncover interesting aspects of this system. To our knowledge, such calculations have not previously been performed in the literature; similar calculations (but for a superconducting puddle in a superconducting matrix) have been reported by Fang *et al.*[3] and Nunner *et al*[4].

We show here that one striking effect of the surrounding metallic matrix is to significantly *weaken d*-wave superconductivity, such that the resulting *d*-wave order parameter within the puddle is less than that of a bulk homogeneous system with the same pairing interaction. Decreasing the size of

the puddle has the effect of decreasing the average *d*-wave order parameter within the puddle. We additionally find a concurrent increase in the average local density of states (LDOS) at zero energy within the puddle when the puddle size is decreased. The behavior of the puddle as its size is decreased is vastly reminiscent of the effect of increasing disorder on bulk *d*-wave superconductivity, and originates entirely from the mixing of the superconducting states within the puddle with the metallic states of the surrounding matrix. Thus, the surrounding metal induces pair-breaking within the puddle, very similar to the effect of disorder[5–7]. Importantly, our calculations confirm that the negative correlation between gap size and filling expected in the mean-field theory also holds for the heterogeneous case (see Fig. 4d in the main text).

In our simulations, we assume that the *d*-wave superconducting puddles are square patches of size $l \times l$. We self-consistently calculate the order parameter from $\Delta_{\boldsymbol{rr}'} = V_{\boldsymbol{rr}'}\langle c_{\boldsymbol{r}\uparrow}c_{\boldsymbol{r}'\downarrow}\rangle$. The pairing interaction $V_{rr'}$ is nonzero only for nearest-neighbor bonds attached to sites within the $l \times l$ patch, and otherwise vanishes. To compute $\Delta_{rr'}$ and the LDOS $\rho(\boldsymbol{r}, \omega)$, we employ an exact real-space Green's function method particularly suited for very large inhomogeneous systems[8,9]. For the calculations reported here, the system size is 100 × 200, which is larger compared to what more traditional exact-diagonalization methods can access. We iterate the calculation until the order parameter is converged, and we assume that we are at $T = 0$. We take the normal-state dispersion (up to next-nearest-neighbor hopping) to be given by the following parameters: $t_1 = 1$, $t_2 = -0.33$, and $\mu = -1.22$ (from this point on we express all energies in units where $t_1 = 1$). The spatially resolved site-centered *d*-wave order parameter plotted throughout this section is obtained by adding the order parameter on all four bonds connected to a single site but assuming a sign difference between the order parameter on bonds along the *x*-direction and that on bonds along the *y*-direction.

We are interested in determining whether *d*-wave superconductivity in puddles behaves differently compared to the bulk case due to the abundance of low-energy states in the nearby metal, and we will tune the size of the puddles (from 5 × 5 to 17 × 17) in particular to isolate the effect of the nearby metal. One expects that the smaller the puddle, the stronger the effect of the metal, since a larger fraction of the puddle is in close proximity to the metal-superconductor boundary. We take the nearest-neighbor pairing interaction strength to be $V_0 = 1.0$ inside the superconducting puddle and $V_0 = 0$ outside it. To provide a baseline for comparisons, we perform the same calculation for a bulk *d*-wave superconductor as well, with the same nearest-neighbor pairing interaction $V_0 = 1.0$ present throughout the entire system. We will frequently express the puddle size in terms of l/ξ_0, where ξ_0 is the coherence length of the superconducting condensate within the puddle; this is to make clearer the effects of miniaturizing the superconducting puddle to dimensions of the order of ξ_0 itself. Note that we have suppressed here the explicit l-dependence of ξ_0, since as it turns out the magnitude of the superconducting order parameter within the puddle, and consequently ξ_0 itself, depends sensitively on l.

Our results are collected together in Fig. S10. In Fig. S10a, we compare the spectral gap (here rather roughly defined as half the peak-to-peak distance in energy, measured from the LDOS) to the *d*-wave order parameter, with both quantities averaged within the puddle. It can be seen that the spectral gap tracks the *d*-wave order parameter closely for the bulk system and for larger puddles (7 × 7 up to 17 × 17, all corresponding to $2 < l/\xi_0 < 10$), although for puddles the spectral gap slightly *overestimates* the *d*-wave order parameter. However, once the puddle size is small enough such that $l/\xi_0 \approx 1$ (as is the case for the 5 × 5 puddle), a gap is no longer visible in the spectrum, even though a nonzero superconducting order parameter remains within the puddle. The strong pair-breaking effects of the surrounding metal are most easily seen in Fig. S10b. Here we plot the average *d*-wave order parameter within the puddle as a function of l/ξ_0 The average order parameter within all seven puddles considered is considerably less than that of the bulk system, and decreases in magnitude as l/ξ_0 is made smaller. Note that when the puddle is made smaller and smaller, the

mixing of metallic states into the superconducting puddle increases since more of the puddle becomes in closer proximity with the superconductor-metal boundary, and hence there is more pair-breaking. Fig. S10c shows the average zero-energy LDOS for seven different puddle sizes. Notice that the zero-energy LDOS of all puddles is much bigger than that of the bulk system, and that it increases as the puddle size is decreased. As with the *d*-wave order parameter, the large zero-energy LDOS is an effect of the mixing of the metallic states into the superconducting puddle, giving the latter a much larger number of low-energy states than one would expect a bulk *d*-wave superconductor to have. The overall trend is succinctly captured by Fig. S10d, which plots together with the *d*-wave order parameter and the zero-energy LDOS both averaged within the puddle, with the variations in both quantities due solely to the puddle size. It can be seen that these two quantities are inversely proportional to each other, with a large *d*-wave order parameter corresponding to a small zero-energy LDOS and vice versa. This is behavior very similar to that expected from disorder acting on a bulk *d*-wave superconductor; one cannot escape the conclusion that the metallic matrix induces pair-breaking effects within the superconducting puddle very similar to that of disorder.

All of these findings are more explicitly demonstrated in Fig. S11, wherein we show plots of the LDOS vs. energy for three puddle sizes (5 × 5, 7 × 7, and 9 × 9, whose l/ξ_0 values are given approximately by 1.2, 2.4, and 3.6, respectively), in addition to the bulk *d*-wave case. We note first that for superconducting puddles, a striking feature of the LDOS is its very large value at $E = 0$ compared to that of the bulk system. One can also notice that for larger puddles, a gap is easily discerned in the spectrum, and coherence peaks are visible but are broader, less well-defined, and shorter in height compared to those of a bulk system. These features become progressively broader as the puddle is shrunk, and more spectral weight accumulates near the Fermi energy, a result of the fact that the average *d*-wave order parameter becomes smaller the tinier the puddles get. However, when the puddle is made sufficiently small such that $l/\xi_0 \approx 1$, such as the 5 × 5 case here, the gap ceases to be visible in the quasiparticle spectrum, and the LDOS resembles that of a normal metal. Nevertheless, there is still a nonzero *d*-wave order parameter present within the puddle.

In sum, we have shown here some of the surprising effects of embedding a *d*-wave superconducting puddle within a metallic matrix. We have demonstrated that the surrounding metallic matrix has a pair-breaking effect on the superconductivity within the puddle, akin to that of disorder, that fills the gap, including at the Fermi level. We have also shown that the smallness of the puddle has a nontrivial effect on the LDOS, with the quasiparticle spectrum within the puddle showing broad signatures of a gap that progressively becomes filled up and washed out the smaller the puddle becomes. The similarity of the pair-breaking effects of the metallic matrix to disorder points to the difficulty of attributing the effects seen in the experiment and detailed in the main text to purely mean-field effects.

Our calculations show that within a mean-field picture, pair-breaking, whether it be due to disorder or the effect of metallic states on a superconducting puddle, naturally leads to an *anticorrelation* between these two quantities. Such a scenario points to the necessity of "beyond-mean-field" physics in resolving the conundrum posed by the experimental results discussed in the main text.

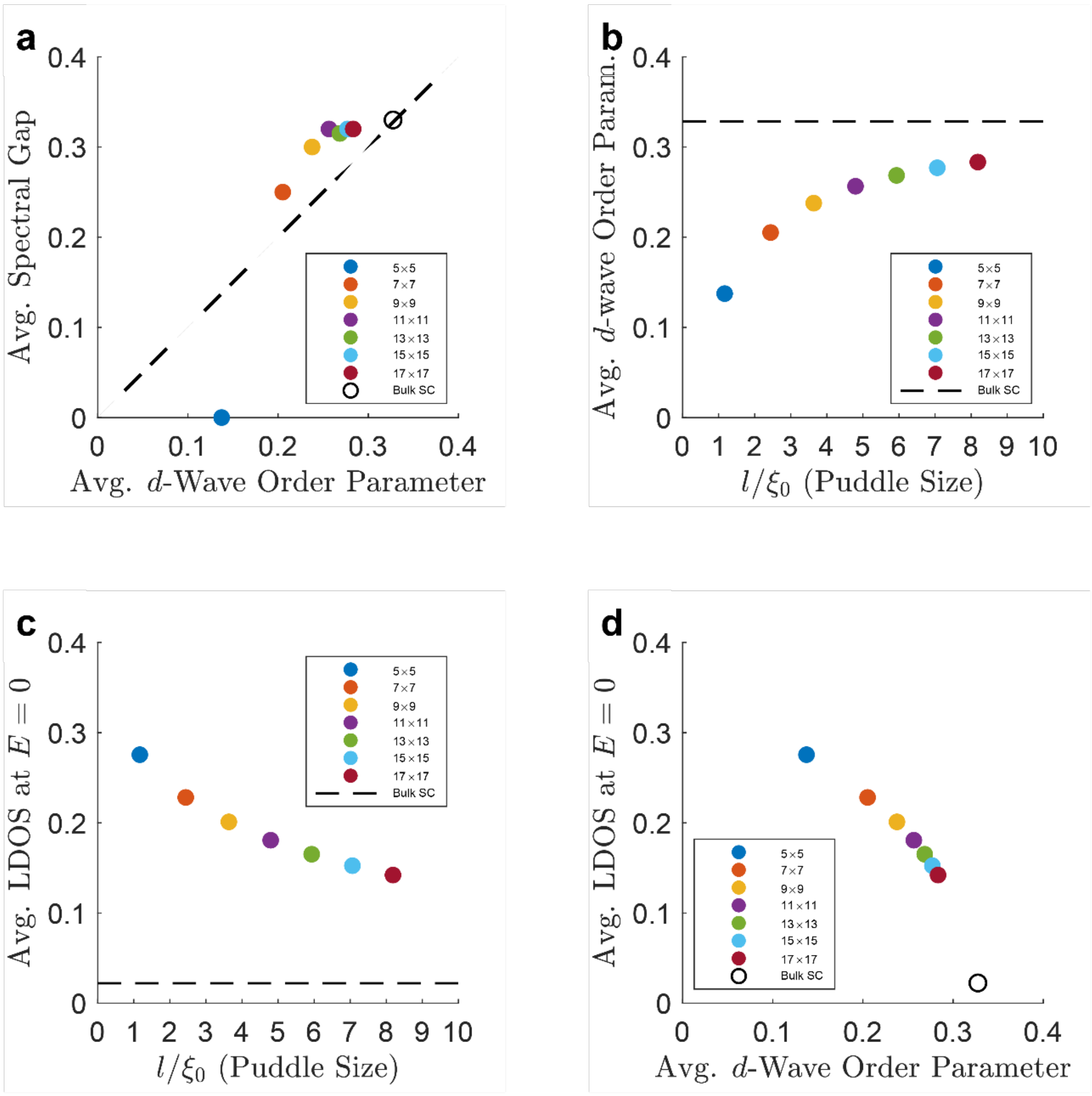

Figure S10: Results for clean superconducting puddles within a metallic matrix of varying size, with V_0 = 1.0. A (top left): plot of the average spectral gap versus the average *d*-wave order parameter, both averaged within the superconducting puddle, with the dashed line indicating where the two quantities are equal. It can be seen that for puddles, the spectral gap is a good indicator of the *d*-wave order parameter (although overestimating it, lying above the dashed line) right until the puddle becomes sufficiently small and $l/\xi_0 \approx 1$ (*e.g.*, 5 × 5), at which point no gap can be seen even though a nonzero superconducting order parameter is present. B (top right): plot of the average of the *d*-wave order parameter within the superconducting puddle versus the puddle size l/ξ_0, with the value for the bulk system shown as a dashed line. C (bottom left): plot of the zero-energy LDOS averaged within the superconducting puddle versus the puddle size l/ξ_0, again with the value for the bulk system shown as a dashed line. D (bottom right): plot of the LDOS at E = 0 versus the *d*-wave order parameter, both averaged inside the superconducting puddle. Evidently, the effect of reducing the puddle size on the superconducting condensate within the puddle is the same as that of increasing the amount of disorder: the *d*-wave order parameter becomes smaller, while the zero-energy LDOS increases.

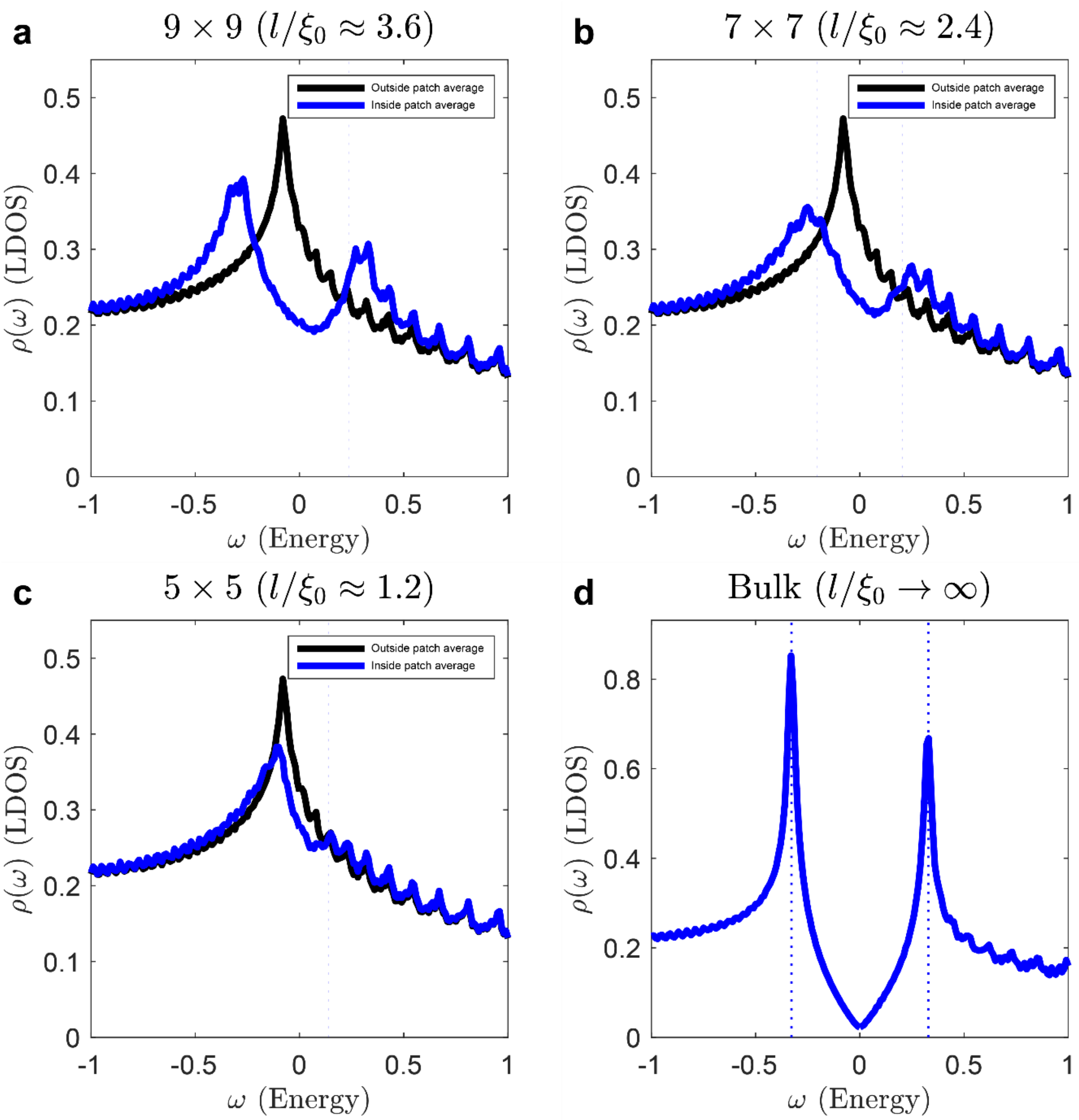

Figure S11: Plots of the LDOS as a function of energy for a *d*-wave superconducting puddle inside a metallic matrix with decreasing puddle size, with V_0 = 1.0. The puddle sizes are 9 × 9, 7 × 7, and 5 × 5 (A-C), corresponding to l/ξ_0 approximately equal to 3.6, 2.4, and 1.2, respectively. Shown are LDOS averages within the puddle (blue) and outside it (black). Also shown for comparison is the average LDOS for a bulk *d*-wave superconductor with the same pairing interaction V_0 = 1.0 (D). The dashed blue lines indicate the average *d*-wave order parameter within the superconducting puddle. Note that as the puddle size becomes smaller, the zero-energy LDOS inside the patch becomes larger, the coherence peaks become smeared out and move to lower energies, and the gap becomes less discernible.

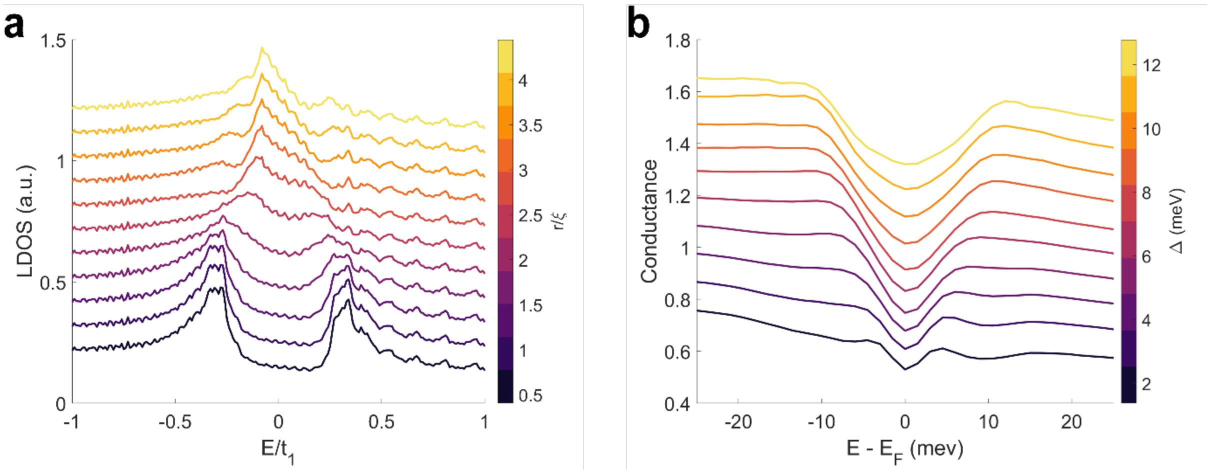

Figure S12: a) Waterfall plot of the spectra calculated for the 9x9 puddle (the same data as presented in main text fig 1f), showing a clear correlation between the gap size going to zero and the coherence peaks disappearing. **b)** Spectra of the OD12K sample binned and averaged according to their gap size *Δ*, as determined by our model. The visibility of the coherence peaks here has no clear relation to the size of the gap. The spectra in **a,b)** have been shifted with respect to eachother for clarity.

V. Rigid band shift in overdoped Bi2201

The doping levels of the superconducting samples are determined using the Presland formula, while the doping level of the OD0K sample is extracted from the rigid band shift measured by ARPES. Below, we describe this procedure more detail.

ARPES measurements on SOD samples show a rigid band shift of the anti-nodal band bottom when the doping is increased (Figure S13). ARPES measurements were performed using the He1α line at 21.2 eV with linear polarization. The sample temperature for all samples was 50K, and the total experimental resolution was set to 6 meV. The k-space cut was along the face of the Brillouin zone (a line spanning the direction (π,π)-(π,0)-(π,-π), indicated in the inset of Fig S13a).

Shown in Fig. S13a as "+" symbols are the positions of the peak maxima of the Energy Distribution Curves (EDC's), extracted after dividing out the resolution broadened Fermi-Dirac distribution from the raw data. The energy position of the band bottom as shown in Fig. S13b is extracted by taking the average of the EDC maxima positions within a small momentum window (indicated in Fig S13a by the two vertical red lines). Using these doping-dependent band bottom energy values, we can determine the doping level of the OD0K crystal by fitting the positions of the superconducting samples and extrapolating the result. We find that the OD0K sample has a doping level of p=0.274±0.008.

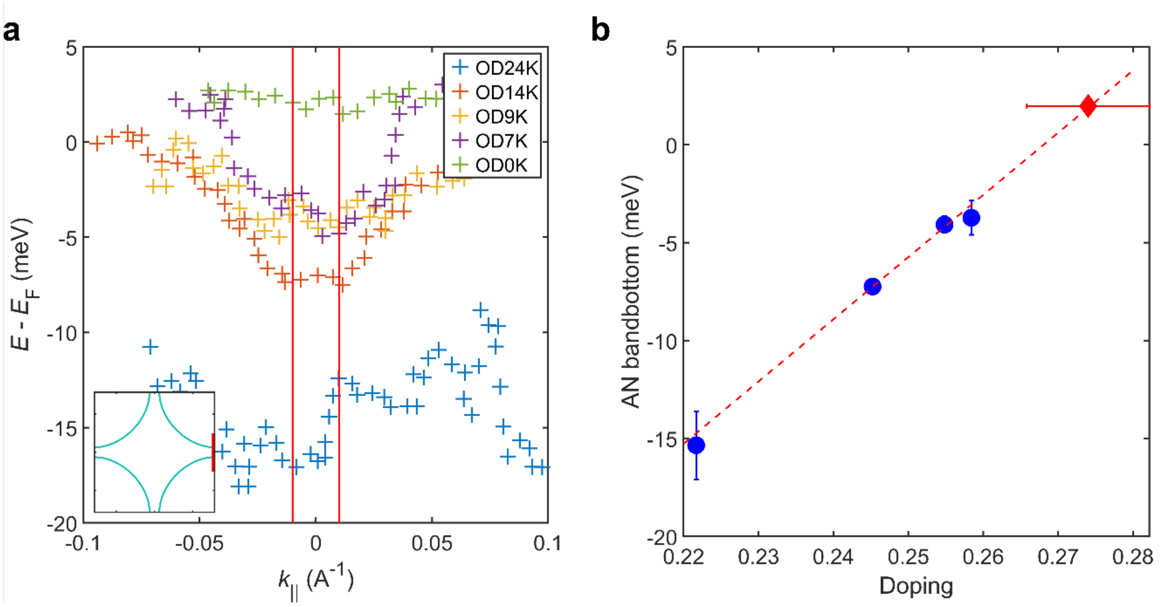

Figure S13: Rigid band shift of the anti-nodal band bottom. The anti-nodal ARPES cut is indicated by the red line on the Fermi surface in the inset to panel (a). In (a) can be seen that the anti-nodal band bottom shifts towards the Fermi level as the samples are progressively overdoped. The energy position of the band bottom is shown in panel (b), and is given by the average position within the red vertical lines in panel (a). The red dashed line in (b) shows a fit through the band bottom energy position for the superconducting samples (blue circles). The fit is then extrapolated to determine the doping level of the non-superconducting sample (red diamond), given its measured band bottom. The error bars in (b) correspond to the calculated standard deviation of the points within the red vertical lines in (a). The red error bar indicates the confidence interval in our fit.

VI. Gap filling and DOS from other experiments

Figure S14 shows the comparison of the gap filling we observe and several other experiments determining the DOS at the Fermi level: optical conductivity[10] (fig S14a), Specific heat measurements[11,12] (fig S14b), and Knight shift[13] (fig S14c). All these probes measure a DOS at Fermi level increasing with overdoping, concomitant with the increased gap filling we observe. We posit that this increased DOS is due to increasingly more Cooper pairs broken by a non-mean field process (see main text).

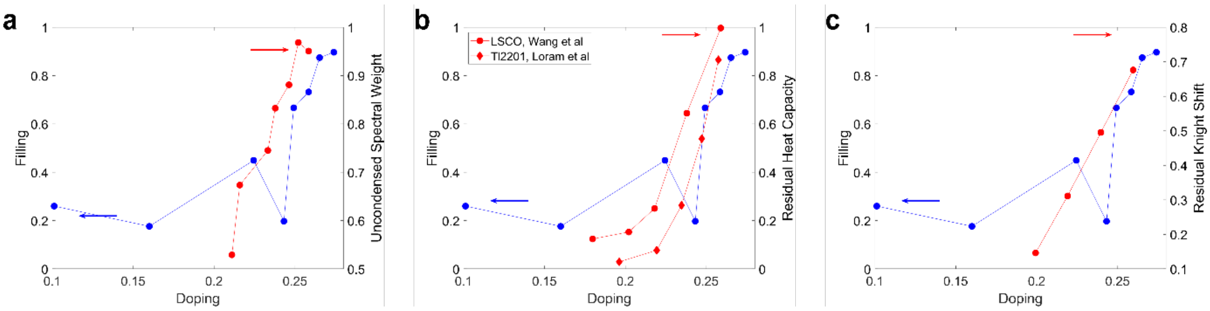

Fig S14: Filling vs spectral weight from optical conductivity below T_c[10] (a), Filling vs residual specific heat[11,12] (b), and Filling vs residual Knight shift below T_c[13] (c).

VII. Pair breaking, Gap filling and competing orders.

In the overdoped cuprates several (fluctuating) orders other than superconductivity have been observed, being two distinct types of charge order and fluctuating ferromagnetism. Here we address their possible relation with our findings.

The charge order of the UD regime continues past optimal doping in the overdoped regime, weakening as the doping increases[14]. This doping dependence is opposite to the behavior of the gap filling, making this an unlikely candidate to participate in the gap filling. The second type of order, sqrt(2) order observed by STM[15]. Research on this phenomenon is ongoing – as mentioned, the relationship to RIXS data is not clear. This CDW is only visible in certain areas, and thus we deduce that it is not directly connected to the phenomenology reported here. In any case, the main points of our paper -- puddle formation, persistent gap, non-mean-field breakdown – remain independent on what exactly this CDW is.

Finally, ferromagnetic fluctuations have been confirmed to exist in overdoped cuprates[16-19], but to what extend these interfere with superconductivity remains unknown. In light of the results presented in this work, one could speculate that small ferromagnetic clusters could reside in the metallic matrix. It would be these small clusters that then give rise to the experimentally observed enhancement of the magnetic susceptibility. Note that this would not necessarily imply competition between these two phases: We observe superconducting islands embedded in a metallic matrix, but the metallic matrix itself is not necessarily caused by ferromagnetic fluctuations, but could also result from for example disorder.

References

1. He, Y. *et al.* Fermi Surface and Pseudogap Evolution in a Cuprate Superconductor. *Science* **344**, 612–616 (2014).

2. Ayres, J. *et al.* Incoherent transport across the strange metal regime of highly overdoped cuprates. *Nature* **595**, 661–666 (2021).

3. Fang, A. C. *et al.* Gap-Inhomogeneity-Induced Electronic States in Superconducting $Bi_2Sr_2CaCu_2O_{8+\delta}$. *Phys. Rev. Lett.* **96**, 017007 (2006).

4. Nunner, T. S., Andersen, B. M., Melikyan, A. & Hirschfeld, P. J. Dopant-modulated pair interaction in cuprate superconductors. *Phys. Rev. Lett.* **95**, 177003 (2005).

5. Franz, M., Kallin, C., Berlinsky, A. J. & Salkola, M. I. Critical temperature and superfluid density suppression in disordered high-Tc cuprate superconductors. *Phys. Rev. B* **56**, 7882 (1997).

6. Lee, P. A. Localized states in a d-wave superconductor. *Phys. Rev. Lett.* **71**, 1887 (1993).

7. Durst, A. C. & Lee, P. A. Impurity-induced quasiparticle transport and universal-limit Wiedemann-Franz violation in d-wave superconductors. *Phys. Rev. B* **62**, 1270 (2000).

8. Sulangi, M. A., Allan, M. P. & Zaanen, J. Revisiting quasiparticle scattering interference in high-temperature superconductors: The problem of narrow peaks. *Phys. Rev. B* **96**, 134507 (2017).

9. Sulangi, M. A. & Zaanen, J. Quasiparticle density of states, localization, and distributed disorder in the cuprate superconductors. *Phys. Rev. B* **97**, 144512 (2018).

10. Mahmood, F., He, X., BoŽović, I. & Armitage, N. P. Locating the Missing Superconducting Electrons in the Overdoped Cuprates $La_{2-x}Sr_xCuO_4$. *Phys. Rev. Lett.* **122**, 027003 (2019).

11. Wang, Y. *et al.* Weak-coupling d -wave BCS superconductivity and unpaired electrons in overdoped La2-x Srx Cu O4 single crystals. *Phys. Rev. B* **76**, (2007).

12. Loram, J. W., Mirza, K. A., Wade, J. M., Cooper, J. R. & Liang, W. Y. The electronic specific heat of cuprate superconductors. *Physica C* **235**–**240**, 134–137 (1994).

13. Ohsugi, S., Kitaoka, Y. & Asayama, K. Temperature dependence of Spin Susceptibility of $La_{2-x}Sr_xCuO_4$ knight shift measurement. *Physica C* **282**–**287**, 1373–1374 (1993).

14. Peng, Y. Y. *et al.* Re-entrant charge order in overdoped $(Bi,Pb)_{2.12}Sr_{1.88}CuO_{6+\delta}$ outside the pseudogap regime. *Nat. Mater.* **17**, 697–702 (2018).

15. Li, X. *et al.* Evolution of Charge and Pair Density Modulations in Overdoped $Bi_2Sr_2CuO_{6+\delta}$. *Phys. Rev. X* **11**, 11007 (2021).

16. Kopp, A. *et al*. Competing ferromagnetism in high-temperature copper oxide superconductors. Proceedings of the National Academy of Sciences, 104(15):6123–6127 (2007).

17. Sonier, J.E. *et al*. Direct search for a ferromagnetic phase in a heavily overdoped nonsuperconducting copper oxide. Proceedings of the National Academy of Sciences, 107(40):17131–17134 (2010).

18. Komiyama Y. *et al*. Magnetic impurity effects on ferromagnetic fluctuations in heavily overdoped $(Bi,Pb)_2Sr_2Cu_{1-y}Fe_yO_{6+\delta}$ cuprates. Journal of the Physical Society of Japan, 90(8):084701 (2021).

19. Sarkar T. *et al*. Ferromagnetic order beyond the superconducting dome in a cuprate superconductor. Science, 368(6490):532–534 (2020).
